# The study of speech and communication in preschool children with autism spectrum disorder

**DOI:** 10.1192/j.eurpsy.2023.2052

**Published:** 2023-07-19

**Authors:** A. Akhmetzyanova, T. Artemyeva

**Affiliations:** 1 Kazan Federal University; 2 Kazan Federal University, Kazan, Russian Federation

## Abstract

**Introduction:**

At preschool age, children develop all the speech functions, including the planning function, which allows them to think through the future speech statement. Researchers find a link between impaired ability for speech forecasting and anxiety disorders, as well as emotional disorders.

**Objectives:**

identifying the specifics of speech forecasting in preschool children with emotional disorders.

**Methods:**

the study involved 48 children: 24 children of preschool age without developmental disorders, 24 children of preschool age with emotional disorders. Empirical examination was carried out using the “Prognostic stories” technique.

**Results:**

Children with autism spectrum disorders often have speech disorders, speech may manifest stereotypes in speech, the appearance of echolalia, many children do not use speech for communication. Among the functional characteristics of forecasting, the cognitive forecasting function has a statistically significant difference (t=4.165<.001). Children with autism spectrum disorders, having difficulties in social interaction, can choose ways of action that are not suitable for the existing situation. Children’s forecasts are usually short-term, invariant, generalized and minimally verbalized.

**Conclusions:**

Indicators of speech-communicative function confirm the presence of difficulties in children with emotional disorders in the ability to verbalize information, however, they have a realistic vision of the future and are focused on following the social norm. This paper has been supported by the Kazan Federal University Strategic Academic Leadership Program.

**Disclosure of Interest:**

None Declared

